# Editorial for the Special Issue on Biomaterials, Biodevices and Tissue Engineering

**DOI:** 10.3390/mi15050604

**Published:** 2024-04-30

**Authors:** Luis Jesús Villarreal-Gómez, José Manuel Cornejo-Bravo, Faruk Fonthal

**Affiliations:** 1 Facultad de Ciencias de la Ingeniería y Tecnología, Universidad Autónoma de Baja California, Tijuana 21500, Mexico; 2 Facultad de Ciencias Químicas e Ingeniería, Universidad Autónoma de Baja California, Calzada Universidad 14418, Parque Industrial Internacional, Tijuana 22300, Mexico; jmcornejo@uabc.edu.mx; 3 Biomedical Engineering Research Group—GBIO, Universidad Autónoma de Occidente, Cali 760030, Colombia; ffonthal@uao.edu.co

Biomaterials, biodevices, and tissue engineering represent the cutting edge of medical science, promising revolutionary solutions to some of humanity’s most pressing health challenges (Figure 1). These interdisciplinary fields are at the forefront of the development of innovative materials, devices, and methodologies to improve healthcare outcomes, enhance patient comfort, and extend human longevity. However, as with any rapidly evolving field, there are both exciting advancements and significant challenges to navigate [1].

One of the most remarkable aspects of biomaterials, biodevices, and tissue engineering is their potential to transform the way we approach healthcare. The possibilities seem limitless, from regenerating damaged tissues and organs to creating implantable devices that seamlessly integrate into the human body. Researchers and clinicians are increasingly leveraging these technologies to develop personalized treatments tailored to individual patients, ushering in an era of precision medicine [2].

In recent years, we have witnessed remarkable progress in the development of biomaterials with enhanced biocompatibility, improved mechanical properties, and bioactivity. These materials serve as the building blocks for a wide range of applications, including tissue scaffolds, drug delivery systems, and implantable devices. Breakthroughs in nanotechnology, 3D printing, and material science have enabled the creation of intricately designed structures at the micro- and nanoscale, mimicking the complexity of native tissues and organs [3].

Biodevices, such as biosensors, wearable health monitors, and implantable medical devices, have also seen significant advancements, revolutionizing disease diagnosis, monitoring, and treatment. These devices offer real-time, non-invasive insights into patients’ health status, empowering individuals to take proactive measures to manage their well-being. Furthermore, integrating biocompatible materials and smart technologies has paved the way for the development of implantable biodevices capable of interfacing with the body’s natural processes [4].

Tissue engineering holds immense promise for addressing the growing demand for organ transplantation and regenerative therapies. By combining cells, biomaterials, and biochemical cues, researchers are striving to create functional tissues and organs in the laboratory for transplantation and disease modeling. While the field has made significant strides in generating simple tissues like skin and cartilage, challenges remain in engineering more complex organs with intricate vascular networks and heterogeneous cell populations [5].

Moreover, biocompatibility, long-term stability, and immune responses remain significant concerns for implanted devices and engineered tissues. Additionally, the scalability and cost-effectiveness of these technologies pose barriers to their widespread adoption in clinical practice. Moreover, ethical considerations surrounding using human-derived cells and tissues in research and transplantation continue to spark debate and raise important questions about consent, equity, and social justice [6].

This editorial summarizes the published studies of the Special Issue on “Biomaterials, Biodevices and Tissue Engineering,” which includes six publications that are discussed as follows:

Solis-Rios et al. [7] present a pioneering application of artificial neural networks (ANNs) to predict the diameter of polyethylene nanofibers, thereby streamlining the parameter adjustment process in electrospinning. Leveraging a dataset of 105 records sourced from the literature, the ANN model demonstrates promising accuracy in predicting nanofiber diameter, with validation against laboratory measurements yielding an average error of 2.29%. The study highlights the potential of machine learning techniques to revolutionize nanofiber production by offering rapid and reliable predictions while emphasizing the need for further validation and expansion of the dataset to ensure the robustness and generalizability of the model. Overall, this research represents a significant step forward in advancing efficiency and reducing costs in producing polyethylene nanofibers, with implications for a wide range of applications [7].

Laubach et al. [8] explores the potential of exopolysaccharides (EPSs) derived from the thermophilic bacterium *Geobacillus* sp. strain WSUCF1, synthesized using cost-effective lignocellulosic biomass, as a foundation for creating 5-fluorouracil (5-FU)-encapsulated films for topical drug delivery. 5-FU, a widely used chemotherapeutic agent, was incorporated into the EPS-based film formulation via a simple self-forming method. The study demonstrates the effectiveness of the 5% 5-FU film against A375 human malignant melanoma cells, with cell viability decreasing to 12% after six hours of treatment. Additionally, the drug release profile exhibited an initial burst release followed by an extended and sustained release of 5-FU. These findings highlight the potential of thermophilic EPSs from lignocellulosic biomass as a versatile platform for delivering chemotherapy drugs and expanding the scope of extremophilic EPS applications [8].

Ashkani et al. [9] discusses the exploration of titanium-based alloys for biomedical applications, focusing on the influence of aluminum and copper additions on mechanical properties. Titanium and its alloys are valued in medical fields due to their corrosion resistance and biocompatibility. The study investigates the addition of non-toxic elements like Mo, Cu, Si, Zr, and Mn to enhance properties such as corrosion resistance and biocompatibility, aiming to develop alloys with improved long-term performance in the human body. Specifically, a Ti-9Mo alloy is modified with aluminum and copper, with copper considered favorable and aluminum potentially harmful to the body. The results show that while the addition of copper decreases the elastic modulus, aluminum increases it. The Ti-Mo-Cu alloy emerges as a promising option due to its favorable properties, suggesting its potential for biomedical applications [9].

The study by Dayob et al. [10] presents an innovative approach to designing self-assembling nanofiber scaffolds for tissue engineering applications, focusing on the modulation of neuronal cell behavior. By utilizing peptide amphiphile (PA) molecules containing multi-functional histidine residues capable of coordinating trace metals (TMs), the authors demonstrate the self-assembly and characterization of PA nanofiber scaffolds, as well as their interaction with the essential microelements zinc (Zn), copper (Cu), and manganese (Mn). Through comprehensive analysis, including the investigation of mammalian cell behavior, reactive oxygen species (ROS), and glutathione levels, the study elucidates the effects of TM-activated PA scaffolds on neuronal PC-12 cells. Particularly noteworthy is the role of Mn (II) in promoting cell–matrix interaction and neuritogenesis, suggesting the potential of histidine-functionalized peptide nanofiber scaffolds to induce regenerative responses. This research offers a promising proof of concept for the development of advanced biomaterials tailored for tissue regeneration and biomedical applications [10].

The study by Durmaz et al. [11] provides a comprehensive overview of the utilization of lignocellulosic bionanomaterials in biosensor applications, addressing the pressing need for sustainable solutions in response to challenges such as rapid population growth, energy demand, and climate change. Lignocellulosic biomass, prized for its abundance, renewability, and cost-effectiveness, offers a compelling alternative for sensor fabrication in various fields, including electronics, communication, biomedical, and tissue engineering. The authors highlight the remarkable properties of lignocellulosic bionanomaterials, such as biodegradability, biocompatibility, and enhanced electrical and thermal conductivity, making them ideal candidates for developing high-impact biosensors. By reviewing current developments and identifying challenges and opportunities, the article underscores the potential of lignocellulosic bionanomaterials to revolutionize biosensor technology and contribute to environmental sustainability [11].

Allu et al. [12] delves into the recent advancements in utilizing cerium oxide nanoparticles (CeO_2_NPs) for wound-healing applications, addressing the complex process of wound healing and the challenges associated with chronic, nonhealing wounds. With a focus on the inflammatory, proliferative, and remodeling phases of wound healing, the authors explore the potential of CeO_2_NPs in reducing inflammation, enhancing hemostasis and proliferation, and scavenging reactive oxygen species. They discuss the mechanisms through which CeO_2_NPs modulate the immune system, promote angiogenesis, and facilitate tissue regeneration, highlighting their antioxidant, anti-inflammatory, and regenerative properties. Furthermore, the review investigates the efficacy of CeO_2_-based scaffolds in creating a conducive environment for wound healing, emphasizing their ability to stimulate wound closure, tissue regeneration, and scar reduction while potentially reducing bacterial infections and enhancing wound-site immunity. However, the authors acknowledge the need for further research to fully understand the safety, efficacy, and long-term impacts of CeO_2_NPs on human health and the environment. Overall, the review highlights the promising potential of CeO_2_NPs in wound healing but also emphasizes the importance of continued investigation to optimize their use and ensure their safety [12].

As we look to the future, collaboration between researchers, clinicians, industry partners, and regulatory agencies will be essential to overcome the discussed challenges and unlock the full potential of biomaterials, biodevices, and tissue engineering. Multidisciplinary approaches that integrate expertise from fields such as biology, engineering, chemistry, and medicine will drive innovation and accelerate the translation of research findings into clinical applications [13]. In conclusion, biomaterials, biodevices, and tissue engineering are promising to revolutionize healthcare and improve patients’ lives. While significant progress has been made, continued investment in research, infrastructure, and education is crucial to address remaining challenges and realize the full transformative potential of these technologies. By harnessing the power of interdisciplinary collaboration and innovation, we can pave the way for a future where personalized, regenerative therapies are accessible to all.

## Figures and Tables

**Figure 1:**
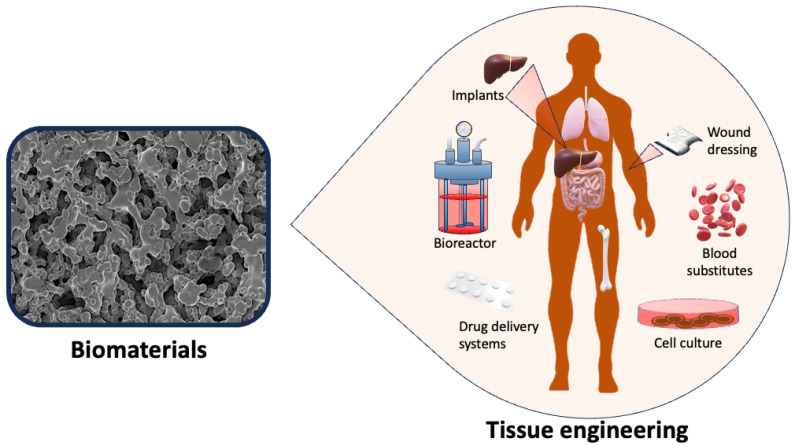
Biomaterials for biomedical applications.
